# Rapidly progressive IgA nephropathy with membranoproliferative glomerulonephritis-like lesions in an elderly man following the third dose of an mRNA COVID-19 vaccine: a case report

**DOI:** 10.1186/s12882-023-03169-3

**Published:** 2023-04-24

**Authors:** Nobuhisa Morimoto, Takayasu Mori, Shingo Shioji, Towako Taguchi, Hatsumi Watanabe, Keigo Sakai, Katsuo Mori, Ayumi Yamamura, Asami Hanioka, Yuichiro Akagi, Tamami Fujiki, Shintaro Mandai, Yutaro Mori, Fumiaki Ando, Koichiro Susa, Soichiro Iimori, Shotaro Naito, Eisei Sohara, Kenichi Ohashi, Shinichi Uchida

**Affiliations:** 1 Department of Nephrology, Graduate School of Medical and Dental Sciences, Tokyo Medical and Dental University, Tokyo, Japan; 2 Department of Comprehensive Pathology, Graduate School of Medical and Dental Sciences, Tokyo Medical and Dental University, Tokyo, Japan; 3 Department of Human Pathology, Graduate School of Medical and Dental Sciences, Tokyo Medical and Dental University, Tokyo, Japan

**Keywords:** IgA nephropathy, Rapidly progressive glomerulonephritis, mRNA COVID-19 vaccine, Hemodialysis, Case report

## Abstract

**Background:**

As messenger RNA (mRNA)-based vaccines for coronavirus disease 2019 (COVID-19) have been administered to millions of individuals worldwide, cases of de novo and relapsing glomerulonephritis after mRNA COVID-19 vaccination are increasing in the literature. While most previous publications reported glomerulonephritis after the first or second dose of an mRNA vaccine, few reports of glomerulonephritis occurring after the third dose of an mRNA vaccine currently exist.

**Case presentation:**

We report a case of rapidly progressive glomerulonephritis in a patient following the third dose of an mRNA COVID-19 vaccine. A 77-year-old Japanese man with a history of hypertension and atrial fibrillation was referred to our hospital for evaluation of anorexia, pruritus, and lower extremity edema. One year before referral, he received two mRNA vaccines (BNT162b2) for COVID-19. Three months before the visit, he received a third mRNA vaccine (mRNA-1273) for COVID-19. On admission, the patient presented severe renal failure with a serum creatinine level of 16.29 mg/dL, which had increased from 1.67 mg/dL one month earlier, prompting us to initiate hemodialysis. Urinalysis showed nephrotic-range proteinuria and hematuria. Renal biopsy revealed mild mesangial proliferation and expansion, a lobular appearance, and double contours of the glomerular basement membrane. Renal tubules had severe atrophy. Immunofluorescence microscopy showed strong mesangial staining for IgA, IgM, and C3c. Electron microscopy exhibited mesangial and subendothelial electron-dense deposits, leading to a diagnosis of IgA nephropathy with membranoproliferative glomerulonephritis-like changes. The kidney function remained unchanged after steroid therapy.

**Conclusions:**

Although the link between renal lesions and mRNA vaccines remains unclear, a robust immune response induced by mRNA vaccines may play a role in the pathogenesis of glomerulonephritis. Further studies of the immunological effects of mRNA vaccines on the kidney are warranted.

**Supplementary Information:**

The online version contains supplementary material available at 10.1186/s12882-023-03169-3.

## Background

As an important measure to combat the coronavirus disease 2019 (COVID-19) pandemic, messenger RNA (mRNA)-based vaccines, including BNT162b2 (Pfizer) and mRNA-1273 (Moderna), have been administered to millions of individuals worldwide [1]. Because of the decline in their effectiveness over time, a third dose of the mRNA vaccine has been recommended in many countries [2, 3]. In Japan, vaccination with a third dose started in December 2021, mainly for the elderly population and healthcare workers [4].

Cases of de novo and relapsing glomerulonephritis after mRNA and adenovirus-vectored COVID-19 vaccination have been reported in the literature [5–8]. Although a causal relationship between mRNA vaccines and renal injury has not been proven, the temporal association suggests a potential link [5, 6]. The induction of a robust immune response by mRNA vaccines and potential cross-reactivity between human severe acute respiratory syndrome coronavirus 2 antibodies and human tissue antigens may increase the risk of autoimmunity, potentially leading to the development of glomerulonephritis [5, 9]. Previous case reports and case series have reported glomerulonephritis after the first or second dose of an mRNA vaccine [5–8]. Only a few reports of glomerulonephritis occurring after the third dose of the mRNA vaccine have been published.

Here, we report a case of rapidly progressive glomerulonephritis in an elderly Japanese man a few months after the administration of the third dose of an mRNA vaccine for COVID-19. The patient presented with severe renal failure that required hemodialysis. A renal biopsy revealed immunoglobulin A (IgA) nephropathy with unique membranoproliferative glomerulonephritis-like changes.

## Case presentation

A 77-year-old man with a history of hypertension and atrial fibrillation was referred to our hospital for evaluation of anorexia, pruritus, and lower-extremity edema. Six years before the visit, his serum creatinine level was 1.37 mg/dL (estimated glomerular filtration rate (eGFR), 40.4 mL/min/1.73m^2^), and urinalysis results were negative for proteinuria and hematuria. The cause of renal impairment was not investigated at the time. One year before the visit, he received two doses of an mRNA vaccine (BNT162b2) for COVID-19 without apparent side effects. Three months before the visit, the patient received a third dose of an mRNA vaccine (mRNA-1273) for COVID-19 without immediate side effects. One month before the presentation, he had a scheduled outpatient visit to the Cardiology Department of our hospital, where his serum creatinine level was 1.67 mg/dL (eGFR, 31.8 mL/min/1.73m^2^). Three weeks before the presentation, he developed edema in the lower extremities and pruritus in his trunk and limbs. His appetite declined two weeks before the visit, and he could not eat for 1 week before the visit. On admission, the patient appeared drowsy. His current medications included bisoprolol (2.5 mg), amlodipine (10 mg), furosemide (20 mg), edoxaban tosylate hydrate (30 mg), and herbal medicine called Shakuyaku-kanzo-to for muscle cramps. The patient did not smoke cigarettes or drink alcohol. He did not report any recent infections. He had never contracted COVID-19 infection previously. On physical examination, he had jugular vein distension, bilateral lower extremity edema, and pruritic rashes on his trunk and limbs. He was afebrile and had a blood pressure of 156/74 mmHg, a pulse rate of 70 beats per minute, and an oxygen saturation of 97% while breathing ambient air. His blood test results showed marked renal impairment (urea nitrogen, 155.3 mg/dL; creatinine, 16.29 mg/dL; eGFR, 2.6 mL/min/1.73m^2^), hyperkalemia, hyperphosphatemia, hypocalcemia, and metabolic acidosis, which are findings equivalent to end-stage kidney disease (ESKD) (Table 1). Chest X-ray radiography and a chest CT scan demonstrated cardiomegaly and bilateral pleural effusions (shown in Supplementary Fig. 1). Echocardiography did not find evidence of the left ventricular wall asynergy or significant valvular lesions. A computed tomography (CT) scan of the abdomen did not show hydronephrosis or enlargement of the bladder, ruling out a post-renal cause (shown in Supplementary Fig. 1). Apparent tumors were absent on the CT scan. The kidneys were slightly enlarged (right, 113 × 46 mm; left, 119 × 57 mm). Fractional excretions of sodium and urea nitrogen were 2.8% and 27.1%, respectively. The inferior vena cava appeared enlarged on the CT scan. Urinalysis revealed the presence of nephrotic-range proteinuria (8.57 g/g creatinine) and hematuria (Table 1).

**Table 1:** Laboratory findings on admission

Blood counts			Reference range	
White blood cell	6,400	/µL	3,300–8,600	/µL
Red blood cell	390	X 10^4^/µL	435–555	X 10^4^/µL
Hemoglobin	11.7	g/dL	13.7–16.8	g/dL
Platelet	13.8	X 10^4^/µL	15.8–34.8	X 10^4^/µL
Reticulocyte	11.3	‰	8.0–22.0	‰
Coagulation			Reference range	
PT-INR	1.12		0.90–1.10	
APTT	27.5	seconds	26.9 ± 25%	seconds
Fibrinogen	433	mg/dL	200–400	mg/dL
FDP	8.3	µg/mL	< 5.0	µg/mL
Venous blood gas			Reference range	
HCO_3_^−^	12	mmol/L	22–26	mmol/L
Blood chemistry			Reference range	
Sodium	145	mEq/L	138–145	mEq/L
Potassium	6.2	mEq/L	3.6–4.8	mEq/L
Chloride	113	mEq/L	101–108	mEq/L
Calcium	6.8	mg/dL	8.8–10.1	mg/dL
Phosphorus	11.4	mg/dL	2.7–4.6	mg/dL
Magnesium	2.3	mg/dL	1.8–2.4	mg/dL
Urea nitrogen	155.3	mg/dL	8.0–20.0	mg/dL
Creatinine	16.29	mg/dL	0.65–1.07	mg/dL
Total protein	6.8	g/dL	6.6–8.1	g/dL
Albumin	2.9	g/dL	4.1–5.1	g/dL
LDH	373	U/L	124–222	U/L
AST	19	U/L	13–30	U/L
ALT	30	U/L	10–42	U/L
ALP	104	U/L	38–113	U/L
γGTP	93	U/L	13–64	U/L
CK	355	U/L	59–248	U/L
CK-MB	7.9	ng/mL	≤ 3.8	ng/mL
Troponin-I	39	pg/mL	≤ 23.4	pg/mL
BNP	751.9	pg/mL	≤ 18.4	pg/mL
C reactive protein	1.28	mg/dL	≤ 0.14	mg/dL
Intact PTH	250.0	pg/mL	10.3–65.9	pg/mL
Total cholesterol	192	mg/dL	142–248	mg/dL
HDL cholesterol	49	mg/dL	38–90	mg/dL
LDL cholesterol	115	mg/dL	65–163	mg/dL
Triglyceride	111	mg/dL	40–234	mg/dL
Blood glucose	109	mg/dL	73–109	mg/dL
HbA1c	5.4	%	4.9–6.0	%
IgG	1504	mg/dL	861–1747	mg/dL
IgA	347	mg/dL	93–393	mg/dL
IgM	66	mg/dL	33–183	mg/dL
Rheumatoid factor	27.7	U/mL	≤ 15.0	U/mL
Anti-nuclear antibody	< 40	fold	< 40	fold
CH50	58	/mL	32–58	U/mL
C3	92	mg/dL	73–138	mg/dL
C4	26	mg/dL	11–31	mg/dL
Cryoglobulin	Negative		Negative	
ASO	23	U/mL	≤ 239	U/mL
ASK	80	fold		
PR3-ANCA	< 1.0	U/mL	< 3.5	U/mL
MPO-ANCA	< 1.0	U/mL	< 3.5	U/mL
Anti-GBM antibody	< 2.0	U/mL	< 3.0	U/mL
Serum electrophoresis	All negative		All negative	
Urine electrophoresis	All negative		All negative	
HBs antibody	Negative		Negative	
HBc antibody	Negative		Negative	
HC antibody	Negative		Negative	
HIV antibody	Negative		Negative	
TP antibody	Negative		Negative	
Urine test			Reference range	
Urine protein	4 +		Negative	
Urine blood	3 +		Negative	
Urine white blood cell	Negative		Negative	
Specific gravity	1.015		1.005–1.030	
Urine pH	5.5		5.0–7.5	
Urine urobilinogen	±		±	
Urine bilirubin	Negative		Negative	
Urine sediment			Reference range	
Red blood cell	> 100	/hpf	< 4	/hpf
White blood cell	10–19	/hpf	< 4	/hpf
Urine chemistry			Reference range	
Urinary protein to creatinine ratio	8.57	g/gCr		
NAG	87.4	U/L	≤ 11.5	U/L
Beta-2 microglobulin	418	µg/L	≤ 289	µg/L

Based on these findings, rapidly progressive glomerulonephritis was suspected. We started sodium zirconium cyclosilicate and sodium bicarbonate administration on the day of admission; additionally, we initiated hemodialysis the following day. His anorexia and edema gradually resolved thereafter. Subsequent serum and urinary screening tests for glomerulopathy revealed an elevated rheumatoid factor level, and the protein fraction test showed a peak in the gamma region (Table 1). Although multiple myeloma, amyloidosis, and monoclonal gammopathy of renal significance were included in our differential diagnoses, serum and urinary immunofixation electrophoresis did not reveal any M-bows. A bone marrow biopsy did not show any monoclonal proliferation of plasma cells, making it unlikely that hematological disease was the cause of his renal dysfunction. The selectivity index was 0.60, suggesting low selectivity. To identify the cause of his renal dysfunction, a percutaneous renal biopsy was performed.

### Renal pathology

**Fig. 1:**
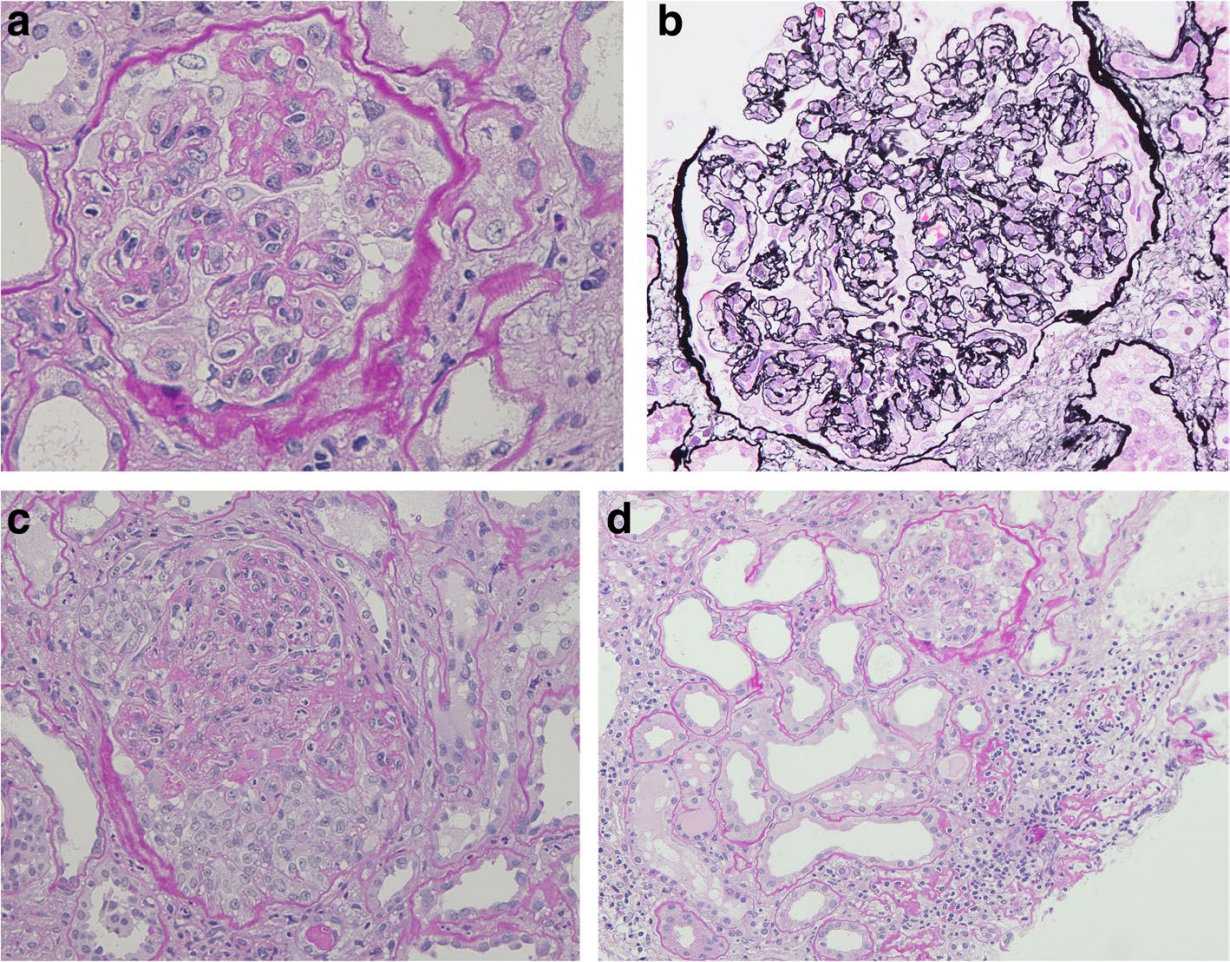
**a** Periodic acid Schiff staining of kidney sections from the patient. Mesangial proliferation and expansion with a lobular appearance are observed (original magnification, × 400). **b** Periodic acid-methenamine-silver staining of kidney sections from the patient. Double contours of the glomerular basement membrane are present (original magnification, × 400). **c** Periodic acid Schiff staining of kidney sections from the patient. The crescent formation is observed at the bottom of the glomerulus (original magnification, × 200). **d** Periodic acid Schiff staining of kidney sections from the patient. Renal tubules showed severe atrophy (original magnification, × 200)

**Fig. 2:**
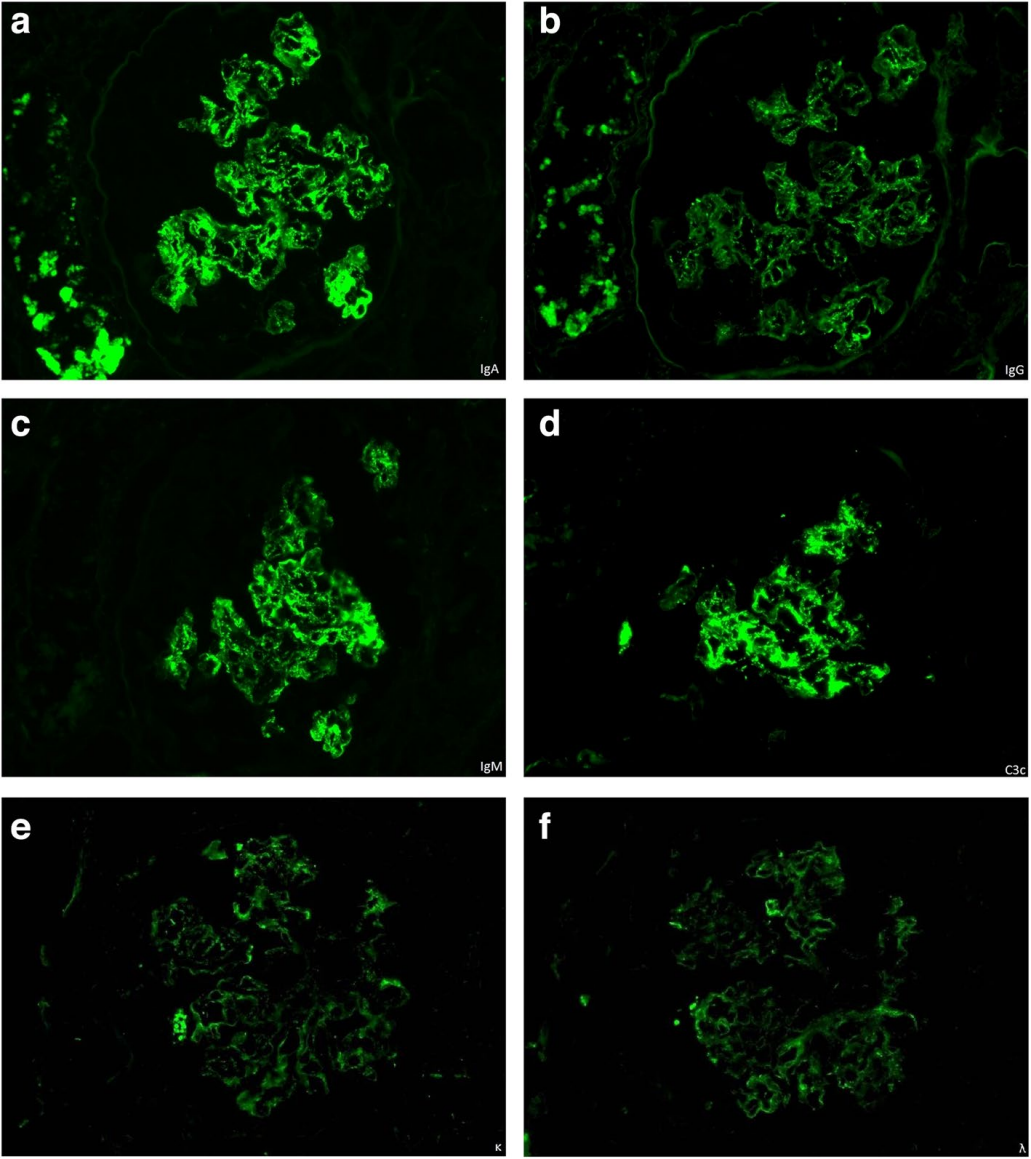
Immunofluorescence images of kidney sections from the patient. Strong mesangial staining of IgA, IgM, and C3 and weak staining of IgG are observed. Weak staining of kappa and lambda light chains is also observed

**Fig. 3:**
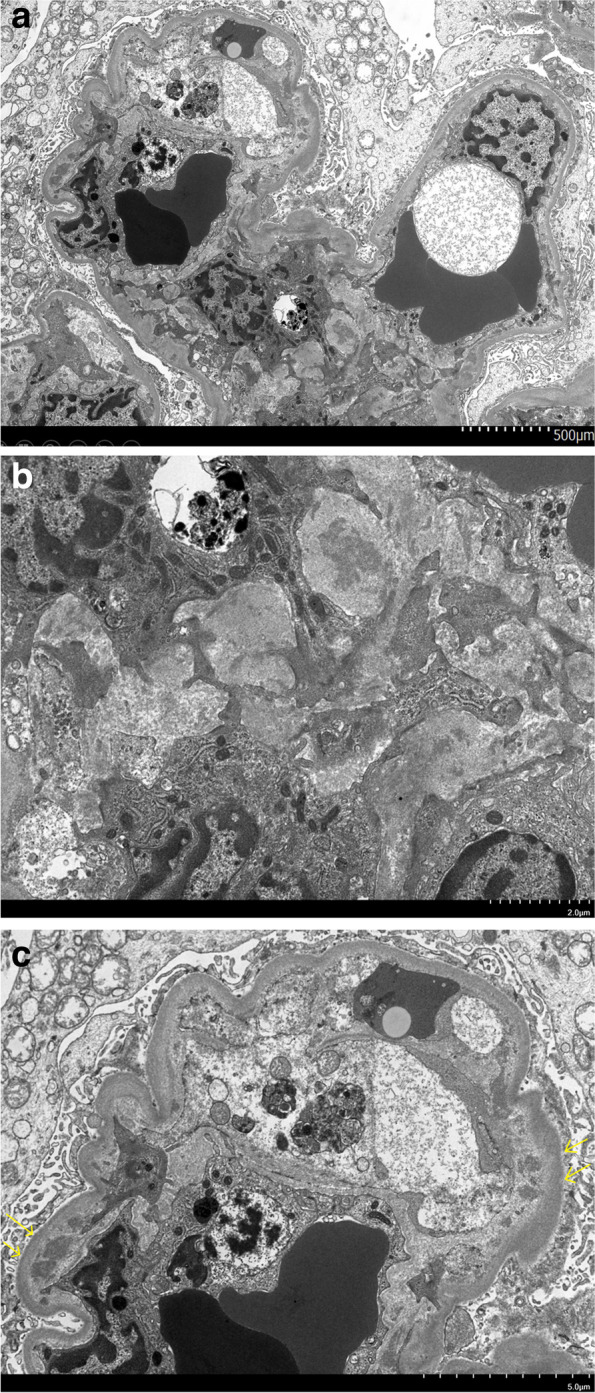
Electron microscopy images of kidney sections from the patient. **a** Mesangial and subendothelial deposits are visible. **b** Mesangial deposits are observed (original magnification, × 3000). **c** Yellow arrows point to subendothelial deposits (original magnification, × 2000)

A total of 13 glomeruli were observed on light microscopy. Seven glomeruli showed global sclerosis, and one glomerulus had segmental sclerosis. Most glomeruli exhibited mild mesangial proliferation and expansion with a lobular appearance, with one glomerulus showing a crescent formation (shown in Fig. 1). Double contours of the glomerular basement membrane were present in some of the glomeruli. The renal tubules showed severe atrophy with mild interstitial fibrosis. Congo-red and direct fast scarlet 4BS staining yielded negative results. Immunofluorescence microscopy demonstrated strong mesangial staining of IgA, IgM, and C3c, and weak mesangial staining of IgG (shown in Fig. 2). Positive staining for IgA and C3c was also observed in the basement membrane. Weak staining of kappa and lambda light chains was also present. Electron microscopy revealed the presence of extensive electron-dense deposits in the mesangial regions, with some deposits in the subendothelial regions (shown in Fig. 3). Podocyte effacement was also observed.

### Differential diagnoses

Considering the presence of proliferative glomerulonephritis with IgA deposits, our differential diagnoses included IgA nephropathy, IgA-dominant postinfectious glomerulonephritis, IgA-dominant membranoproliferative glomerulonephritis (MPGN), and IgA-proliferative glomerulonephritis with monoclonal immunoglobulin deposits (PGNMID). Given the absence of monoclonality of immunoglobulins on immunofluorescence, the possibility of PGNMID was unlikely. Postinfectious glomerulonephritis was deemed less likely because of the absence of a recent infection history, normal complement levels, and lack of subepithelial deposits or humps on electron microscopy [10]. Based on the strong staining of IgA, IgM, and C3c in the mesangial regions, the most probable diagnosis was IgA nephropathy. Paraneoplastic IgA nephropathy was less likely as imaging studies and a bone marrow biopsy found no apparent tumor lesions, and his symptoms resolved after starting hemodialysis.

### Final diagnosis

IgA nephropathy with MPGN-like changes, grade III(A/C) M1E1S1T1C1.

### Treatment and clinical course

Suspecting IgA nephropathy and MPGN based on light microscopy and immunofluorescence findings, we started a 3-day intravenous methylprednisolone pulse followed by 0.8 mg/kg/day (50 mg) of oral prednisolone. Once we found the electron microscopy findings consistent with IgA nephropathy, we administered two additional courses of methylprednisolone pulse therapy and oral steroid therapy. His renal function remained largely unchanged after 1 month of steroid therapy. For hemodialysis vascular access, we created an arteriovenous fistula in the right forearm; however, its maturation was poor. Another attempt to create a fistula was unsuccessful. Eventually, a long-term vascular catheter was inserted in the right jugular vein. The dose of prednisolone was reduced to 15 mg/day, and the patient was discharged without any symptoms on Day 51.

## Discussion and conclusions

We report a case of rapidly progressive renal dysfunction in an elderly patient following three doses of mRNA vaccines. Our patient exhibited three unique features. First, the rate of decline in renal function was very rapid. Second, renal pathology revealed mesangial IgA deposition with MPGN-like changes. Third, he received the third dose of an mRNA COVID-19 vaccine 3 months before presentation to our hospital.

The patient’s clinical course and pathological findings supported the diagnosis of rapidly progressive IgA nephropathy, defined as IgA nephropathy with > 50% decline in the eGFR within 3 months after excluding reversible causes and other causes of RPGN [11]. Notably, our patient's renal function decline rate was much faster than that reported in previous studies [12, 13]. For example, a Japanese study of rapidly progressive IgA nephropathy showed a mean initial serum creatinine level of 1.5 mg/dL [12]. One potential reason for the rapid decline in renal function was the concomitant presence of significant tubular damage, as indicated by severe tubular atrophy on renal pathology and marked elevation of renal tubular markers. One contributing factor was decreased renal perfusion, as suggested by the reduced fractional excretion of urea nitrogen. We considered the possibility of dehydration because of anorexia before admission, intravascular volume reduction caused by hypoalbuminemia resulting from nephrotic syndrome, and heart failure. However, he reported that he had drunk fluids before admission. Additionally, it is questionable whether his mild hypoalbuminemia significantly reduced intravascular volume. While his heart failure could cause renal impairment, the patient was hemodynamically stable without apparent left ventricular wall asynergy or significant valvular lesions, suggesting the possibility of the cardio-renal syndrome was less likely. Another factor that may cause tubular damage is the administration of new medications; however, the patient did not recall the use of new medications during recent months. We were unable to identify the cause of significant tubular damage.

Uniquely, our patient’s renal pathology showed mesangial IgA deposition and electron-dense deposits in the mesangial and subendothelial regions with double contours of the basement membrane. Relatively few previous reports have presented similar pathological findings [14, 15]. One case report described a 55-year-old man who presented with rapidly progressive renal failure and proteinuria in the nephrotic range and demonstrated IgA and C3 deposits on immunofluorescence and extensive subendothelial electron-dense deposits with double contours of the basement membrane on electron microscopy [14]. While the possibility of both IgA-dominant MPGN and IgA nephropathy with an MPGN-like pattern existed, the final diagnosis was IgA-dominant MPGN due to the lack of mesangial/paramesangial deposits. Another report of two children showed deposition of IgA and C3 along the capillary wall of glomeruli with type-I MPGN histological findings [15]. One previous study of 244 patients with IgA nephropathy found no cases with MPGN-like changes, whereas subepithelial and/or subendothelial deposits were observed in 23% of cases [16]. Considering these prior studies, IgA nephropathy with an MPGN-like pattern, as observed in our patient, is likely to be very rare.

Although the most probable diagnosis was IgA nephropathy, the possibility of IgA-dominant postinfectious glomerulonephritis, which occurs most frequently in elderly men and can cause acute renal failure with hematuria and proteinuria [10], could not be excluded. Some previous reports showed atypical cases of postinfectious glomerulonephritis, which did not entail hypocomplementemia or a recent infection history [10, 17, 18], similar to our patient’s presentation. While the overlapping clinical and pathological features of IgA nephropathy, IgA-dominant postinfectious glomerulonephritis, and IgA-dominant MPGN posed a diagnostic challenge [14, 17], we considered IgA nephropathy with MPGN-like changes most likely given the presence of mesangial and subendothelial deposits, strong staining for IgA, normal complement levels, and lack of an overt infection [10].

One potential trigger of glomerulonephritis in our patient was the administration of the third dose of an mRNA vaccine. Recent reports have described de novo and relapsing IgA nephropathy following the first or second dose of an mRNA vaccine [5, 6]. Cases of rapidly progressive IgA nephropathy within 3 months of COVID-19 vaccination have also been reported [5, 19]. Two elderly men developed rapidly progressive IgA nephropathy after the first dose of the adenovirus vector vaccine (Astra Zeneca) [5]. Both patients presented with renal failure and nephrotic syndrome, and one patient required dialysis treatment. One adolescent without a pertinent medical history developed rapidly progressive IgA nephropathy within 24 h after the first dose of the mRNA vaccine [19]. She required hemodialysis temporarily; however, her renal function improved after the initiation of steroid therapy. Another potential trigger of our patient’s glomerulonephritis was the administration of different types of mRNA vaccines (BNT162b2 and mRNA-1273). However, the number of published reports on the topic has been very few, precluding us from evaluating its possibility. One retrospective cohort study in Canada demonstrated an increased risk of glomerulonephritis relapse after the second or third dose of the COVID-19 vaccine [20]. While 10% of patients in the study had received one BNT162b2 dose and one mRNA-1273 dose, the study did not evaluate the effect of multiple vaccine types on the risk of relapse. Whether the administration of different types of mRNA vaccines increases glomerulonephritis risk merits further investigation.

Regarding a plausible mechanism that links mRNA vaccines and IgA nephropathy, some researchers hypothesized that Toll-like receptor (TLR) signaling induced by mRNA vaccines might be implicated in the formation of aberrantly glycosylated IgA1, which could be deposited in the glomeruli and cause glomerulonephritis [21, 22]. One previous study showed that the increased expression of TLR-7, which recognizes single-stranded RNAs in the endosome [23, 24], led to greater production of galactose-deficient IgA1 and inflammatory cytokines in the kidneys [25]. Therefore, single-stranded RNA from mRNA vaccines may activate TLR-7 and have a role in the development of IgA nephropathy. Another plausible mechanism relates to a hypothesized postinfectious glomerulonephritis mechanism: single-stranded RNA from vaccines acts as a superantigen, which binds to the major histocompatibility class II molecules on antigen-presenting cells and T cell receptors, leading to T cell activation and significant inflammation [10, 26]. Further studies are warranted to elucidate the mechanistic link between glomerulonephritis and mRNA vaccines.

Previous studies indicated a poor renal prognosis for patients with IgA nephropathy who had a rapidly progressive course, tubular atrophy, or MPGN-like changes [13, 27, 28]. One multicenter cohort study of 113 Chinese patients with crescentic IgA nephropathy showed that approximately 70% of patients developed end-stage renal disease within 5 years, regardless of immunosuppressive therapy [13]. The initial serum creatinine level strongly predicted ESKD, as > 95% of patients with initial serum creatinine > 6.8 mg/dL developed ESKD during follow-up. Another cohort study of 858 Japanese patients with IgA nephropathy demonstrated a poor response to steroid therapy in patients with renal tubular atrophy [27]. One study of 27 patients with IgA deposition on immunofluorescence and MPGN features found a poor renal prognosis [28]. Our patient’s renal prognosis was likely poor because of his high initial serum creatinine level (16.29 mg/dL) and severe renal tubular atrophy found in renal pathology. Although we decided to initiate steroid therapy based on the rapidly progressive course and a mild degree of interstitial fibrosis, his renal function remained largely unchanged after 1 month of steroid treatment, suggesting a poor renal prognosis.

In summary, we present a case of rapidly progressive IgA nephropathy with MPGN-like lesions in an elderly man after the third dose of an mRNA COVID-19 vaccine. While we do not have evidence proving causality, a strong immune response induced by the mRNA vaccines might be involved in the development of significant renal damage. The accumulation of similar case reports may help elucidate the potential link between mRNA vaccines and glomerulonephritis.

## Supplementary Information

**Additional file 1: Supplementary Fig. 1.** (a) Chest computed tomography (CT) scan. Bilateral pulmonary effusion was present. (b) Abdominal CT scan. Hydronephrosis was absent. Apparent tumor lesions were absent.

## Data Availability

The datasets used and analyzed during the current study are available from the corresponding author upon reasonable request.

## Abbreviations

COVID-19: Coronavirus disease 2019 (COVID-19)
mRNA: Messenger RNA
IgA: Immunoglobulin A
eGFR: Estimated glomerular filtration rate
ESKD: End-stage kidney disease
PT-INR: Prothrombin time-international normalized ratio
APTT: Activated partial thromboplastin time
FDP: Fibrinogen degradation products
LDH: Lactate dehydrogenase
AST: Aspartate aminotransferase
ALT: Alanine aminotransferase
ALP: Alkaline phosphatase
γGTP: Gamma-glutamyl transferase
CK: Creatine kinase
BNP: Brain natriuretic peptide
PTH: Parathyroid hormone
HDL: High-density lipoprotein
LDL: Low-density lipoprotein
HbA1c: Hemoglobin A1c
ASO: Antistreptolysin O
ASK: Anti-streptokinase
ANCA: Antineutrophil cytoplasmic antibody
GBM: Glomerular basement membrane
HB: Hepatitis B
HC: Hepatitis C
HIV: Human immunodeficiency virus
TP: Treponema pallidum
hpf: High power field
NAG: N-acetyl-beta-D-glucosaminidase
CT: Computed tomography
MPGN: Membranoproliferative glomerulonephritis
PGNMID: Proliferative glomerulonephritis with monoclonal immunoglobulin deposits
TLR: Toll-like receptor
