# Supplementary figures and images for: Rapidly progressive IgA nephropathy with membranoproliferative glomerulonephritis-like lesions in an elderly man following the third dose of an mRNA COVID-19 vaccine: a case report
Source: BMC Nephrol. 2023 Apr 24;24:108. doi: 10.1186/s12882-023-03169-3 (PMC10123559; doi:10.1186/s12882-023-03169-3)

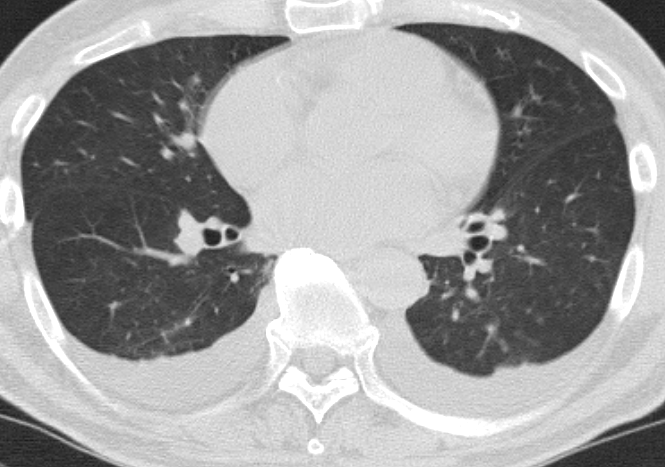

Supplement: Supplementary file 1 — Additional file 1: Supplementary Fig. 1. (a) Chest computed tomography (CT) scan. Bilateral pulmonary effusion was present. (b) Abdominal CT scan. Hydronephrosis was absent. Apparent tumor lesions were absent. [file 12882_2023_3169_MOESM1_ESM.zip › Supplementary_Figure_1a_finalR2.png]

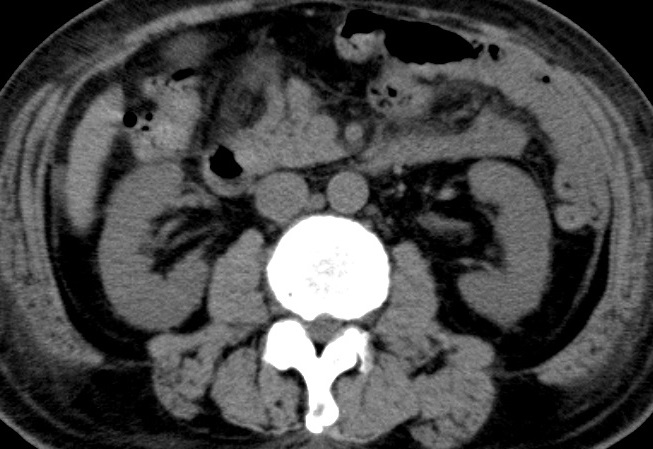

Supplement: Supplementary file 1 — Additional file 1: Supplementary Fig. 1. (a) Chest computed tomography (CT) scan. Bilateral pulmonary effusion was present. (b) Abdominal CT scan. Hydronephrosis was absent. Apparent tumor lesions were absent. [file 12882_2023_3169_MOESM1_ESM.zip › Supplementary_Figure_1b_finalR2.jpg]
